# Seronegative Propylthiouracil‐Induced Reversible Bilateral Sensory Neural Hearing Loss Without Systemic Vasculitis: A Case Report and Review of Literature

**DOI:** 10.1002/ccr3.73722

**Published:** 2026-10-29

**Authors:** Adane Alemayehu Abera, Genetu Dagnaw Alemayehu, Zelalem Tadesse Wondimu, Behaylu Tesfamaryam Hagos, Mequannet Tesfaw Addis, Mohammedsefa Arusi Dari

**Affiliations:** ^1^ Department Internal Medicine, Asrat Weldeyes Health Sciences College Debre Berhan University Debre Berhan Ethiopia; ^2^ Department of Otolaryngology Head and Neck Surgery Addis Ababa University Addis Ababa Ethiopia

**Keywords:** adverse drug reaction, case report, graves' disease, ototoxicity, propylthiouracil, sensorineural hearing loss

## Abstract

Propylthiouracil (PTU) is a thioamide drug used in the management of hyperthyroidism in selected clinical settings. While PTU is associated with well‐known adverse effects such as hepatotoxicity, agranulocytosis, and antineutrophil cytoplasmic antibody (ANCA)‐associated vasculitis, ototoxicity is an exceptionally rare complication. We present a case of reversible bilateral sensorineural hearing loss following PTU therapy in the absence of systemic vasculitis. A 42‐year‐old Ethiopian woman with newly diagnosed Graves' disease was commenced on PTU 100 mg orally three times daily and propranolol 40 mg daily. After approximately two months of PTU therapy, the patient developed gradual onset bilateral hearing loss which worsened within 2 weeks before she presented for evaluation accompanied by tinnitus, without vertigo or prior history of ear disease, noise exposure, or use of other ototoxic medications. Physical examination revealed diffuse, nontender goiter and bilateral eighth cranial nerve palsy, with normal otoscopic findings. Audiometric evaluation demonstrated bilateral, symmetric, down‐sloping high‐frequency sensorineural hearing loss, with normal hearing thresholds at low and mid frequencies and moderate‐to‐severe hearing loss at 4–8 kHz. Laboratory work‐up excluded infectious, autoimmune, and metabolic causes, including negative ANCA and antinuclear antibody serologies. PTU was discontinued and a short course of oral prednisolone (40 mg daily for five days) was initiated. Hearing began to improve within days and normalized completely by day ten, confirmed by follow‐up audiometry. This case highlights PTU‐induced ototoxicity as a rare but reversible adverse event, even in the absence of systemic vasculitis or ANCA positivity. Clinicians should maintain a high index of suspicion for auditory symptoms in patients on PTU therapy. Early recognition and prompt drug withdrawal are essential to prevent permanent hearing impairment. Based on the WHO‐UMC causality assessment criteria, this adverse reaction was classified as probable/likely.

**Abbreviations**
ANA: antinuclear antibody
ANCA: antineutrophil cytoplasmic antibody
FNAC: fine needle aspiration cytology
HCG: human chorionic gonadotropin
HIV: human immunodeficiency virus
MD: medical doctor
MPO‐ANCA: myeloperoxidase antineutrophil cytoplasmic antibody
PTU: propylthiouracil
SNHL: sensory nural hearing loss
T3: triiodothyronine
T4: thyroxine
TSH: thyroid stimulating hormone
VDRL: venereal diseases research laboratory
WHO‐UNC: World Health Organization‐Uppsala Monitoring Centre

## 1 Introduction

Propylthiouracil (PTU) is a thioamide medication that inhibits thyroid peroxidase and the peripheral conversion of thyroxine (T4) to triiodothyronine (T3), making it effective in reducing thyroid hormone levels [1, 2]. It remains a cornerstone in the management of hyperthyroidism, particularly in a specific clinical context such as the first trimester of pregnancy, thyroid storm, or in patients who are intolerant to methimazole [3, 4].

Despite its therapeutic utility, PTU is associated with a range of well‐documented adverse effects. The most recognized include hepatotoxicity, agranulocytosis, and antineutrophil cytoplasmic antibody (ANCA)‐associated vasculitis [5, 6]. Other less common complications, such as lupus‐like syndrome and glomerulonephritis, have also been reported [7, 8, 9]. However, ototoxicity is an exceptionally rare complication of PTU therapy. A review of the literature reveals only a few isolated case reports describing hearing impairment in the context of PTU use (Table 1), the majority of which occurred in patients with concurrent ANCA‐associated vasculitis [10, 11, 12].

**TABLE 1:** Literature summary table for previously reported cases of PTU‐associated ototoxicity.

Author	Year	Duration of PTU exposure	ANCA‐status	Systemic vasculitis	Hearing outcome
Smith & Spaulding	1972	Months	Not reported	No	Partial recovery
Maguchi et al.	2001	Years	MPO‐ANCA positive	Yes	Variable
Thamprajamchit et al.	2004	Years	Positive	Yes	Partial recovery
Hiruma et al.	2003	Years	Positive	Yes	Improved
Raja et al.	2010	Weeks	Negative	No	Recovered
Tanaka et al.	2018	Years	Positive	Yes	Variable
Tanabe et al.	2021	Months	Positive	Yes	Partial recovery
Present case	2025	2 months	Negative	No	Complete recovery

Ototoxicity is broadly defined as damage to the structure or function of the inner ear, including the cochlea, vestibular apparatus, and auditory nerve, resulting from exposure to pharmacological agents [13, 14]. While classically associated with medications like aminoglycosides, loop diuretics, and platinum‐based chemotherapeutics, drug‐induced ototoxicity can also arise from other mechanisms, including immune‐mediated injury or vasculitic changes within the cochlear vasculature [15, 16]. Clinically, it often presents as sensorineural hearing loss (SNHL), tinnitus, or balance disturbances [17, 18].

Given the rarity of PTU‐associated ototoxicity, particularly in the absence of systemic vasculitis, this adverse effect may be underrecognized. To our knowledge, this is the first documented case of PTU‐induced reversible bilateral SNHL without systemic vasculitis in Ethiopia. This case report aims to highlight the importance of considering ototoxicity in patients on PTU therapy and to apply the World Health Organization‐Uppsala Monitoring Centre (WHO‐UMC) criteria to establish causality.

## 2 Case Presentation

### 2.1 Patient History/Physical Examination

A 42‐years‐old female was diagnosed with Graves' disease following clinical features of hyperthyroidism, including weight loss, palpitations, hot intolerance and tremors on June, 2025. Physical examination revealed tachycardia (heart rate 123 beats per minute and regular) and diffuse, nontender, firm anterior neck swelling. Baseline otoscopic examination and hearing assessment were normal.

After approximately 2 months of treatment, the patient developed gradual onset progressive bilateral hearing loss associated with tinnitus. She denied vertigo, ear discharge, head trauma, prior ear disease, noise exposure, or use of other ototoxic medications, like aminoglycosides or loop diuretics. On examination, tympanic membranes were intact bilaterally. Bedside hearing tests (finger rub and whispered voice) suggested reduced hearing acuity. Neurological examination was normal. There were no signs of rash, arthralgia, renal impairment, respiratory symptoms, or other systemic manifestations.

### 2.2 Investigations

Initial laboratory investigations revealed suppressed thyroid‐stimulating hormone (TSH < 0.09 uIU/mL), elevated free thyroxine levels (fT4 = 29.07 pmol/L), and elevated free T3 level (free T3 = 7.38 pmol/L), and FNAC from thyroid glands shows benign goiter.

Laboratory Investigations after starting treatment (PTU 100 mg po TID and propranolol 40 mg po daily) revealed normal thyroid stimulating hormone (TSH = 1.6 uIU/mL), normal thyroxine hormone (free T4 = 17 pmol/L), normal triiodothyronine hormone (free T3 = 4.9 pmol/L). Other laboratory results, including complete blood count, random blood sugar, renal function and hepatic function tests were within normal limits and urine HCG negative. Additionally, an autoimmune panel (including antinuclear antibody (ANA), rheumatoid factor, and MPO‐ANCA) and screening for infections (including HIV, syphilis (VDRL), hepatitis B virus surface antigen, and hepatitis C virus antibody) were negative. Audiometric evaluation demonstrated bilateral, symmetric, down‐sloping high‐frequency sensorineural hearing loss, with normal hearing thresholds at low and mid frequencies and moderate‐to‐severe hearing loss at 4–8 kHz (Figure 1).

**FIGURE 1:**
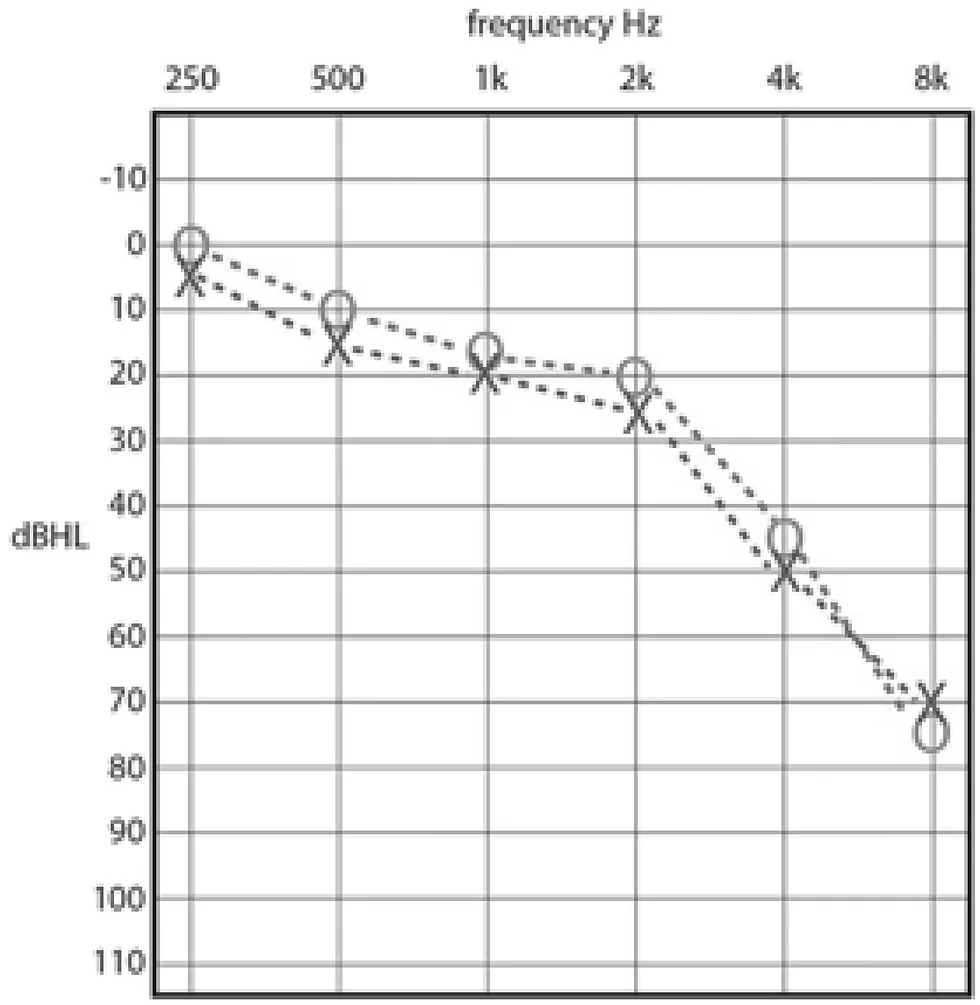
Baseline pure‐tone audiogram demonstrating bilateral, symmetric, down‐sloping high‐frequency sensorineural hearing loss, with relatively preserved low‐ and mid‐frequency thresholds and moderate‐to‐severe hearing loss at 4–8 kHz.

### 2.3 Diagnosis

Given the temporal association with PTU therapy and exclusion of alternative causes, PTU‐induced ototoxicity is considered as the probable/likely cause with a Naranjo adverse drug reaction probability scale score of 6 (Table 2) and WHO‐UMC adverse drug reaction assessment criteria (Table 3), and later bilateral sensory hearing loss was confirmed by audiometry (Figure 1).

**TABLE 2:** Naranjo adverse drug reaction probability scale showing our patient probability score.

Question	Yes	No	Don't know	Score
1. Are there previous conclusive reports on this reaction?	+1	0	0	+1
2. Did the adverse event appear after the suspected drug was administered?	+2	−1	0	+2
3. Did the adverse reaction improve when the drug was discontinued or a specific antagonist was administered?	+1	0	0	+1
4. Did the adverse reaction reappear when the drug was readministered?	+2	−1	0	0
5. Are there alternative causes (other than the drug) that could on their own have caused the reaction?	−1	+2	0	+2
6. Did the reaction reappear when a placebo was given?	−1	+1	0	0
7. Was the drug detected in the blood (or other fluids) in concentrations known to be toxic?	+1	0	0	0
8. Was the reaction more severe when the dose was increased or less severe when the dose was decreased?	+1	0	0	0
9. Did the patient have a similar reaction to the same or similar drugs in any previous exposure?	+1	0	0	0
10. Was the adverse event confirmed by any objective evidence?	+1	0	0	0
Total score				+6

**TABLE 3:** WHO‐UMC causality assessment criteria with interpretation showing our patient.

Criteria	Case findings	Interpretation
Time relationship	Hearing loss occurred weeks after starting PTU	Compatible
Alternative causes excluded	No toxic drugs, autoimmune disease, infection, noise exposure, or metabolic cause identified	Strongly supportive
De‐challenge effect	Hearing improved after stopping PTU	Positive
Rechallenge	Not performed (not required for “probable”)	Not required
Known reaction	Rare but documented in case reports	Plausible
Final WHO‐UMC category	—	Probable/Likely

### 2.4 Management and Outcome

PTU was discontinued immediately. A short course of oral prednisolone (40 mg po daily for five days) was initiated to address a potential inflammatory or hypersensitivity mechanism. Hearing began to improve within 3–4 days of drug withdrawal and completely normalized by day 10. Follow‐up audiometry confirmed full recovery (Figure 2). For definitive management of graves diseases and to avoid re‐exposure to antithyroid drugs, she subsequently undergoes thyroidectomy.

**FIGURE 2:**
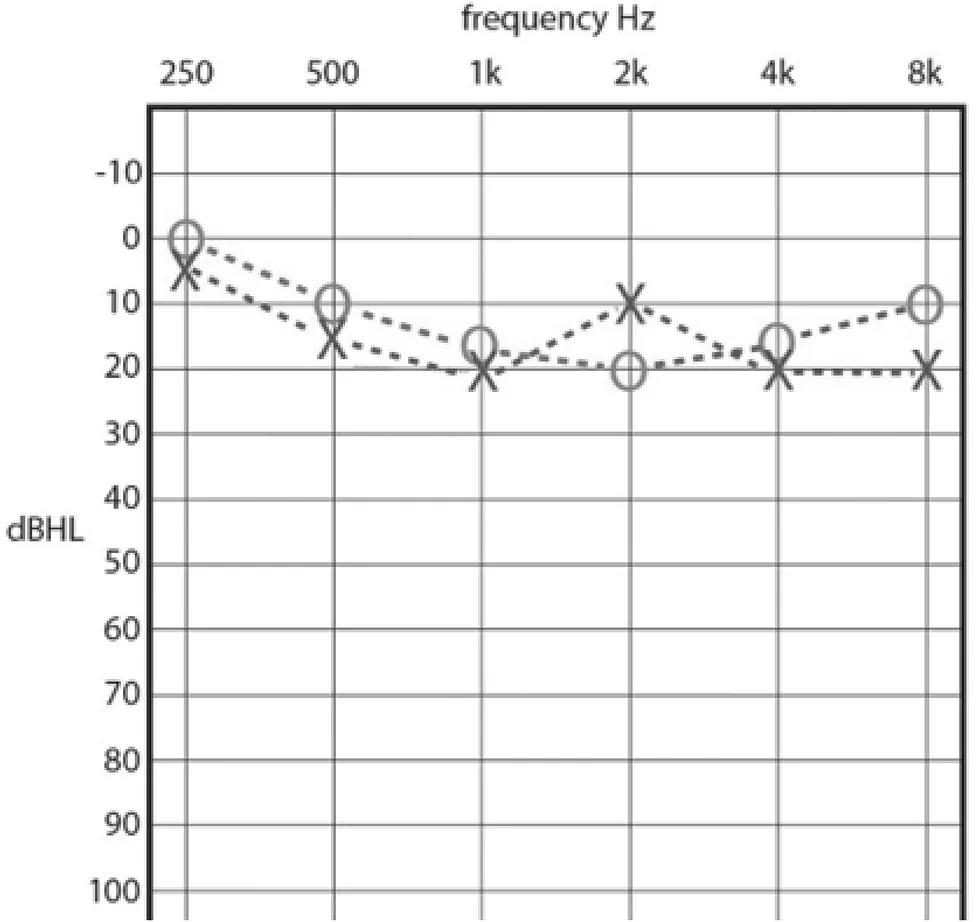
Follow‐up pure‐tone audiogram demonstrating recovery of bilateral hearing thresholds to the normal range following discontinuation of PTU and corticosteroid treatment.

## 3 Discussion

We present a rare case of a 42‐year‐old female with Graves' disease who developed progressive, bilateral sensorineural hearing loss and tinnitus after two months of PTU therapy. The absence of other identifiable causes, the temporal relationship between drug initiation and symptom onset, and the rapid and complete recovery following drug cessation strongly support the diagnosis of PTU‐induced ototoxicity. Based on the Naranjo Adverse Drug Reaction Probability Scale (score of 6) and the WHO‐UMC causality assessment system, this adverse reaction is classified as probable/likely.

The pathophysiology of PTU‐induced ototoxicity remains poorly understood but is likely multifactorial. The most frequently proposed mechanism is an immune‐mediated process, often linked to PTU‐induced ANCA‐associated vasculitis (AAV). In this model, immune complex deposition or direct antibody‐mediated inflammation can damage the stria vascularis and sensory hair cells of the cochlea, leading to SNHL [19, 20]. Several case reports support this mechanism. For instance, Thamprajamchit et al. described a 22‐year‐old woman with prolonged PTU use who developed bilateral SNHL, vertigo, and systemic symptoms in the setting of MPO‐ANCA positivity [11]. Similarly, Sano et al. and Hiruma et al. reported cases of progressive hearing loss with systemic AAV after years of PTU therapy, where recovery was slow and required immunosuppression [12].

Our case, however, presents a distinct clinical picture. The onset of symptoms occurred acutely after only two months of therapy, which is significantly shorter than the multi‐year exposure reported in most vasculitic cases. Furthermore, our patient lacked any systemic symptoms (e.g., fever, arthralgia, rash) and tested negative for both ANCA and ANA. This profile diverges from the classic PTU‐induced AAV presentations and suggests a different, potentially non‐ANCA‐mediated mechanism. This could involve a localized hypersensitivity reaction within the cochlea, direct but reversible cytotoxicity of PTU or its metabolites on outer hair cells, or subclinical vasculitis confined to the cochlear microvasculature that was insufficient to trigger systemic serological markers [19, 20, 21].

The reversibility observed in our patient is a key feature. The rapid improvement in hearing within days of discontinuing PTU and the short course of corticosteroids contrasts with the often slow and incomplete recovery seen in ANCA‐positive cases [11, 12, 18, 22]. This favorable outcome supports the hypothesis of an inflammatory or functional insult rather than permanent structural damage. It underscores the critical importance of early recognition and prompt drug withdrawal, which can prevent irreversible hearing loss [23, 24].

This case has important clinical implications. First, it demonstrates that PTU‐induced ototoxicity can occur early in the treatment course and in the complete absence of systemic vasculitis or ANCA positivity. Therefore, a high index of suspicion must be maintained for any auditory symptoms in patients taking PTU, regardless of treatment duration or the presence of other systemic features. Second, new‐onset tinnitus or hearing loss in this context should prompt immediate drug discontinuation. Third, while the role of corticosteroids is not definitively established for ANCA‐negative cases, a short course may be considered to address potential inflammatory components and possibly accelerate recovery.

The primary limitation of this report is its single‐case nature, which limits the generalizability of our findings. Additionally, while a rechallenge was not performed for ethical reasons, the strong temporal association and positive de‐challenge provide robust evidence for causality.

This case expands the recognized clinical spectrum of PTU‐induced ototoxicity by demonstrating that it can present as an early, isolated, ANCA‐negative, and fully reversible condition. It serves as a critical reminder for clinicians to maintain vigilance for this rare but potentially debilitating adverse effect.

## 4 Conclusion

This case expands the clinical spectrum of PTU‐induced ototoxicity by demonstrating an early‐onset presentation, ANCA negative serology, isolated PTU‐ototoxicity in the absence of systemic vasculitis, rapid and complete reversibility.

It highlights a rare instance of reversible bilateral sensorineural hearing loss associated with PTU use. The absence of other etiologies and improvement following cessation of PTU support a causal link; Naranjo adverse drug reaction probability scale and WHO‐UMC criteria further support a Probable/Likely causal relationship. Awareness of PTU's potential ototoxicity enables early diagnosis, timely management, and prevention of permanent auditory impairment. Clinicians should maintain a high index of suspicion for auditory symptoms in patients treated with PTU, and prompt drug withdrawal may prevent irreversible hearing loss.

## Author Contributions

**Adane Alemayehu Abera:** conceptualization, investigation, writing – original draft, methodology, validation, writing – review and editing, visualization, formal analysis, data curation, supervision, resources. **Genetu Dagnaw Alemayehu:** writing – review and editing, visualization, formal analysis, supervision, validation, methodology, conceptualization, data curation. **Zelalem Tadesse Wondimu:** methodology, supervision, visualization, writing – review and editing, investigation, validation. **Behaylu Tesfamaryam Hagos:** investigation, validation, data curation, supervision, writing – review and editing. **Mequannet Tesfaw Addis:** validation, investigation, supervision, methodology. **Mohammedsefa Arusi Dari:** supervision, validation, writing – review and editing.

## Funding

The authors have nothing to report.

## Consent

Written informed consent was obtained from the patient for publication of this case report and any accompanying images.

## Conflicts of Interest

The authors declare no conflicts of interest.

## Data Availability

All information and data supporting the findings of this case report are included within the manuscript.
